# Changes in Liver Cell DNA Methylation Status in Diabetic Mice Affect Its FT-IR Characteristics

**DOI:** 10.1371/journal.pone.0102295

**Published:** 2014-07-14

**Authors:** Benedicto de Campos Vidal, Flávia Gerelli Ghiraldini, Maria Luiza S. Mello

**Affiliations:** Department of Structural and Functional Biology, Institute of Biology, University of Campinas (Unicamp), Campinas, São Paulo, Brazil; University of Quebect at Trois-Rivieres, Canada

## Abstract

**Background:**

Lower levels of cytosine methylation have been found in the liver cell DNA from non-obese diabetic (NOD) mice under hyperglycemic conditions. Because the Fourier transform-infrared (FT-IR) profiles of dry DNA samples are differently affected by DNA base composition, single-stranded form and histone binding, it is expected that the methylation status in the DNA could also affect its FT-IR profile.

**Methodology/Principal Findings:**

The DNA FT-IR signatures obtained from the liver cell nuclei of hyperglycemic and normoglycemic NOD mice of the same age were compared. Dried DNA samples were examined in an IR microspectroscope equipped with an all-reflecting objective (ARO) and adequate software.

**Conclusions/Significance:**

Changes in DNA cytosine methylation levels induced by hyperglycemia in mouse liver cells produced changes in the respective DNA FT-IR profiles, revealing modifications to the vibrational intensities and frequencies of several chemical markers, including ν_as_ –CH_3_ stretching vibrations in the 5-methylcytosine methyl group. A smaller band area reflecting lower energy absorbed in the DNA was found in the hyperglycemic mice and assumed to be related to the lower levels of –CH_3_ groups. Other spectral differences were found at 1700–1500 cm^−1^ and in the fingerprint region, and a slight change in the DNA conformation at the lower DNA methylation levels was suggested for the hyperglycemic mice. The changes that affect cytosine methylation levels certainly affect the DNA-protein interactions and, consequently, gene expression in liver cells from the hyperglycemic NOD mice.

## Introduction

Non-obese diabetic (NOD) mice are a useful experimental model in which an expressive amount of females spontaneously develop a form of autoimmune diabetes that closely resembles human diabetes [1]. In NOD mice, a combination of apparently normal alleles at numerous recessive loci is associated with insulin-dependent diabetes. Each of these alleles contributes a small degree of susceptibility to the disease [1], [2]. In the NOD hyperglycemic mouse liver, gene expression-profiles and cellular metabolism are also affected [3]–[6].

Lower cytosine methylation levels have been found in DNA from liver cells in NOD mice under hyperglycemic conditions when the bulk genome is considered [7]. This epigenetic change may be associated with increased chromatin accessibility to MNase digestion and decreased chromatin compactness in hyperglycemic NOD mice [8]. Because Fourier transform-infrared (FT-IR) profiles of dry DNA samples are affected differently by certain DNA characteristics, such as base composition, single-strandedness and histone binding [9], the methylation status of DNA from NOD mice is expected to affect the DNA FT-IR profile. The FT-IR notation indicates that a Fourier transform algorithm is necessary to convert the raw data into a spectrum, which is performed using modern technology for IR microspectroscopy, wherein a beam of IR light is passed through a sample at all wavenumbers, thus revealing specific absorption peaks.

The effects of methylation on the backbone structure of DNA sequence models in solution at different levels of methylation have been investigated with FT-IR, wherein the spectral regions that are sensitive to the base-sugar conformation were emphasized [10]. Dry synthetic oligonucleotide samples with different methylation patterns were studied using FT-IR in the 1700–800 cm^−1^ spectral range and Raman spectroscopy, which revealed gradual changes as a function of differing methylation content [11]. However, FT-IR data have not been collected from dry DNA samples at different levels of methylation and analyzed in the 3600–2800 cm^−1^ spectral range, which is sensitive to –NH and –NH_2_ group stretching vibrations [12]–[16], ν_as_ and ν_s_ C-H stretching vibrations in the 5-methylcytosine methyl group [14], [17], and general -CH_3_ and –CH_2_ groups [12], [17]–[19] and may include hydrogen bonds [12].

In the present study, differences in the FT-IR signatures of liver DNA previously demonstrated with different methylated cytosine levels [7] were investigated in NOD mice that were of the same age and under normoglycemic as well as hyperglycemic conditions.

## Results

**Figure 1:**
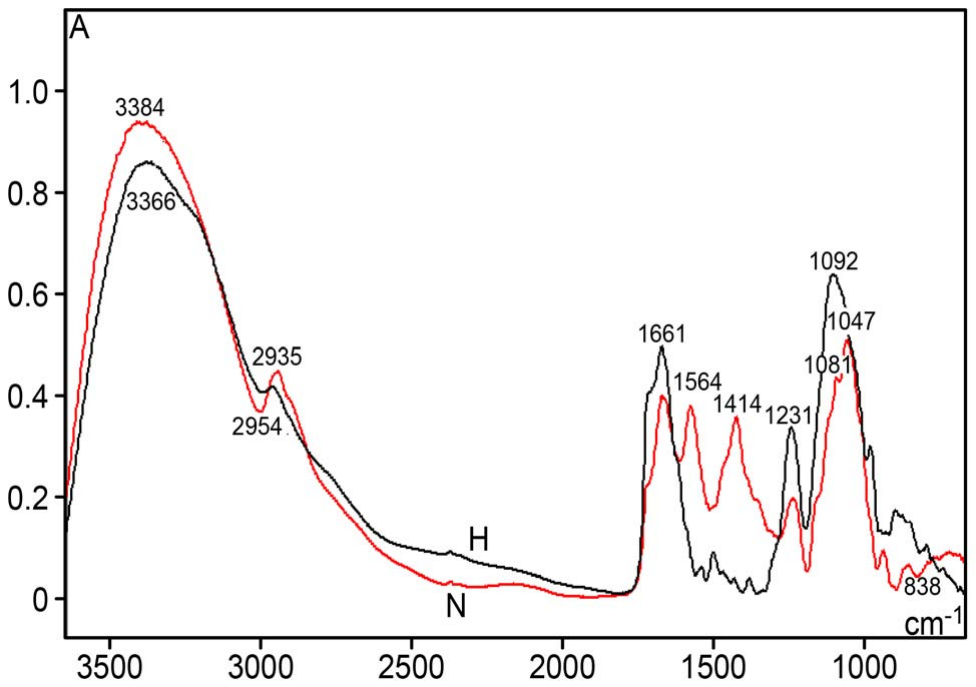
FT-IR spectral profiles for the liver DNA from NOD mice. Normoglycemic mice (N), red line; hyperglycemic mice (H), black line; spectral range: 3600–700 cm^−1^. X axis, wavenumbers in cm^−1^; Y axis, absorbances (A).

**Figure 2:**
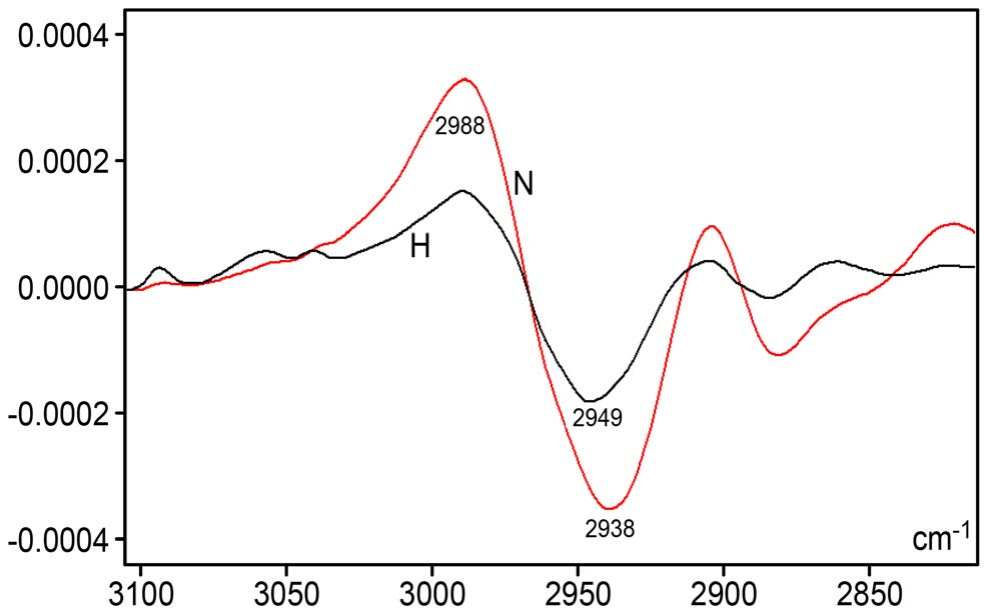
Savitzky-Golay's second-derivative spectra for the liver DNA from NOD mice. Detail for the IR spectral window in the 3100–2800 cm^−1^ range. Normoglycemic mice (N), red line; hyperglycemic mice (H), black line; X axis, wavenumbers in cm^−1^; Y axis, second derivative. Software: Grams/AI 8.0; 2nd derivative degree: 2, points: 31.

**Figure 3:**
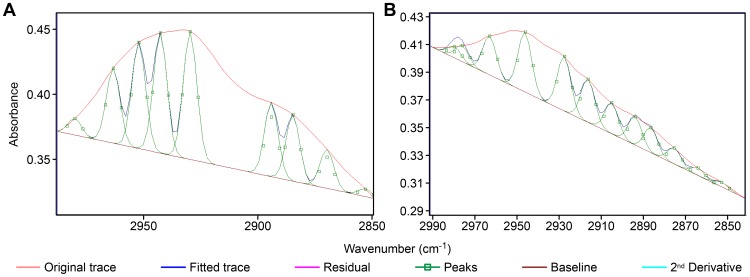
FT-IR spectral profiles after peak fitting for the liver DNA from NOD mice. Normoglycemic mice, A; hyperglycemic mice, B. Spectral range: 2990–2850 cm^−1^. Software: Grams/AI 8.0; function: Gaussian; sensitivity: low.

A comparison of the FT-IR profiles between hyperglycemic and normoglycemic mice revealed differences in the spectral range from 3600–2800 cm^−1^, 1700–1500 cm^−1^ and 1450–700 cm^−1^ (the fingerprint region) (Fig. 1). The most elevated peak, especially assigned to –NH and –NH_2_ group stretching vibrations [12]–[16], and also possibly to DNA hydrogen bonding [12], occurred at 3384 cm^−1^ for normoglycemic mice and at ∼3366 cm^−1^ for hyperglycemic mice. The absorbance intensity at ∼2987–2849 cm^−1^, which was assigned to the ν_as_ and ν_s_ C-H stretching vibrations in 5-methylcytosine methyl groups [14], was more elevated in the DNA spectral profile from hyperglycemic mice and was confirmed using the second-derivative for this spectral window (Fig. 2). In addition, the area under this band peak was >2-fold smaller for hyperglycemic mouse DNA compared with normoglycemic mouse DNA (Table 1). Such a DNA band peak for normoglycemic mice was resolved into nine components after peak fitting using the FT-IR equipment software (sensitivity: low) (Fig. 3A); the most prominent peak was estimated at 2931 cm^−1^. In the hyperglycemic mouse DNA, the band peak was resolved into 12 components (Fig. 3B); the most prominent peak was estimated at ∼2944 cm^−1^.

**Table 1:** Numerical statistics for the FT-IR –CH_3_ band peak in the DNA from NOD mouse liver cell nuclei.

NOD mouse states	DNA –CH_3_ band		Normoglycemic/hyperglycemic –CH_3_ area ratio	Band center of mass
	Peak (cm^−1^)	Area units		cm^−1^
Normoglycemic	2931	7.47	2.69	2924
Hyperglycemic	2945	2.78		2930

Wavenumber edges: 2987 and 2849 cm^−1^. Software: Grams; function: Gaussian; sensitivity: low.

**Figure 4:**
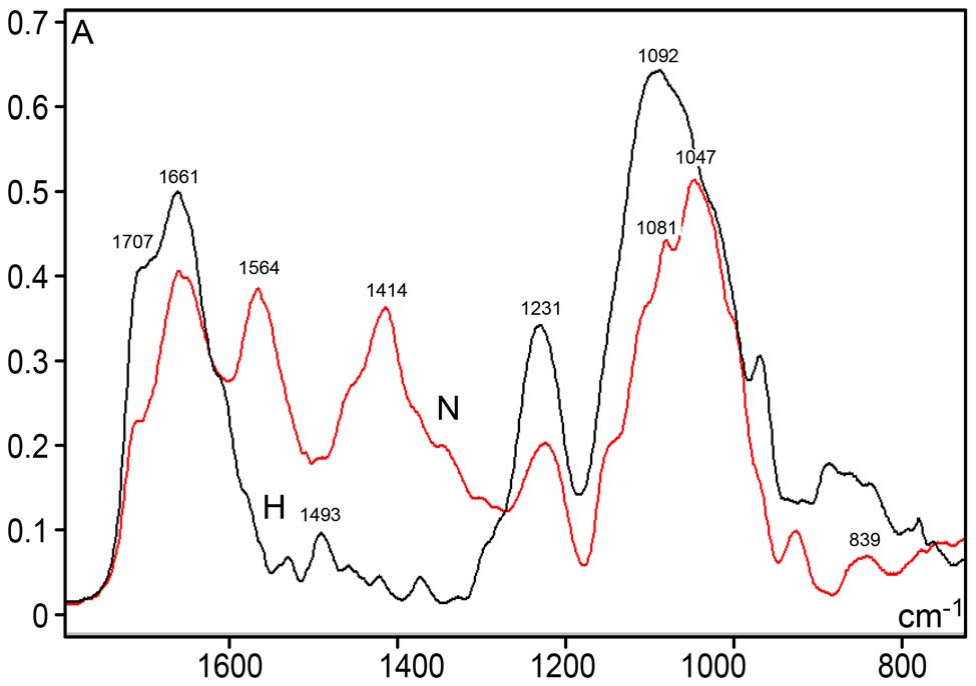
FT-IR spectral profiles for the liver DNA from NOD mice. Detail of the IR spectral window in the 1800–730 cm^−1^ range. Normoglycemic mice (N), red line; hyperglycemic mice (H), black line. X axis, wavenumbers in cm^−1^; Y axis, absorbances (A).

**Figure 5:**
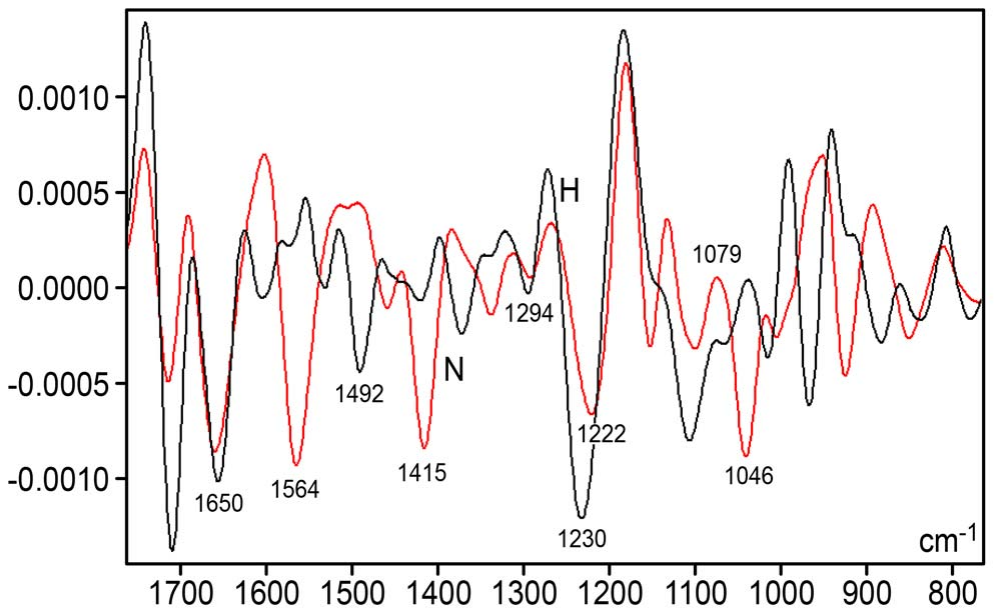
Savitzky-Golay's second-derivative spectra for the liver DNA from NOD mice. Detail for the IR spectral window in the 1750–800 cm^−1^ range. Normoglycemic mice (N), red line; hyperglycemic mice (H), black line; X axis, wavenumbers in cm^−1^; Y axis, second derivative. Software: Grams/AI 8.0; 2nd derivative degree: 2, points: 31.

A shoulder at 1707 cm^−1^ and a peak at 1661 cm^−1^ were notably more elevated in the DNA spectrum for the hyperglycemic mice (Figs. 1 and 4). When the second-derivative was determined for the 1800–730 cm^−1^ spectral window, the peak corresponding to 1661 cm^−1^ shifted to 1650 cm^−1^ for hyperglycemic mouse DNA (Fig. 5).

The absorbance intensity at ∼1564 cm^−1^ decreased with lower methylation (Figs. 1, 4). A small peak at 1493 cm^−1^ was only evident in the DNA from the hyperglycemic NOD mouse liver cells, but an absorption peak at 1294 cm^−1^ was not clearly evident in the DNA from either the normoglycemic or hyperglycemic mice (Figs. 4 and 5). Studies have reported that peaks at the 1493 and 1294 cm^−1^ frequencies are contributed by cytosine only [20] or by both cytosine and guanine [21] (or the band at 1492 cm^−1^ is specifically assigned to cytosine and guanine [22]).

In the spectral regions related to PO_2_^−^ antisymmetric (1220–1226 cm^−1^) and symmetric (1090–1080 cm^−1^) stretching, pentose ring vibrations and the main S-type sugar markers [10], [21], the vibrational intensities increased with the decreasing methylation observed in the hyperglycemic mice (Figs. 4 and 5). The peak that corresponds to the vibration intensity of the antisymmetric stretching in the DNA PO_2_^−^ groups was lower than the groups' symmetric stretching in both normoglycemic and hyperglycemic NOD mice, which has been extensively reported for several other material types. The second derivative fitted to the IR profile revealed that the frequencies related to the DNA PO_2_^−^ antisymmetric stretching in the normoglycemic and hyperglycemic mice slightly differ (1222 cm^−1^ and 1230 cm^−1^, respectively (Fig. 5). For the PO_2_^−^ symmetric stretching frequency, the DNA from the normoglycemic mice exhibited a peak at ∼1081 cm^−1^ (Figs. 4 and 5), and the DNA peak from the hyperglycemic mice appeared at 1092 cm^−1^ in the original profile (Fig. 4), but was resolved from second-derivative spectra at ∼1100 cm^−1^ (Fig. 5).

An elevated peak at 1047 cm^−1^ and a low peak at 839 cm^−1^ were evident in the normoglycemic mouse DNA spectral profile (Figs. 4 and 5). At the spectral region with frequencies <900 cm^−1^, absorptivity was more intense for the hyperglycemic mouse DNA.

## Discussion

The findings herein reveal that the hyperglycemia-induce changes in the methylation status of DNA from mouse liver cells produced differences in the corresponding DNA FT-IR profiles obtained using microspectroscopic procedures. Further, the vibrational intensities and frequencies of several chemical markers are modified, including ν_as_ and ν_s_ C-H stretching vibrations from 5-methylcytosine methyl groups of [14], [17]. Based on reports that the area under an absorption band peak in IR spectroscopy is related to the absorbed energy [16], [18], [23], this study showing a smaller band area for the hyperglycemic NOD mouse DNA –CH_3_ groups suggests a lower abundance of cytosine methylation in the liver cell nuclei of these mice, which is consistent with a report by Damasceno et al. [7]. The different number of peaks and the position of the most prominent peak resulting from the peak-fitting procedure indicate changes in the chemical environment energy levels due to changing DNA methylation levels, which occur with increasing glycemia levels in NOD mice.

The literature has assigned a band peak such as that exhibited by the NOD mouse DNA FT-IR signature in the ∼2987–2849 cm^−1^ spectral range to the ν_as_ of –CH_3_ and –CH_2_ groups, especially in proteins [9], [19]. However, in the present case this peak is assumed to be primarily due to ν_as_ and ν_s_ C-H vibrations in the 5-methylcytosine –CH_3_ group [14], [17]. A peak in this IR spectral region, although not as evident as in the presently studied NOD mouse DNA samples, has also been observed in commercially available, protein-free pure DNA samples, examined with an all-reflecting objective (ARO) [16]. In addition, the 260/280 nm absorbance ratio obtained using a spectrophotometer and the protocol for isolating DNA from the NOD mouse liver indicates that protein contaminants were not present in these samples [24].

For DNA analysis in the lower frequency FT-IR region, it may be considered that ARO may be not as adequate as an ATR objective [16]. Additionally, most DNA vibrational group data from the literature that was used for comparison are from samples in solution environments. However, when studying the DNA spectral profiles for the hyperglycemic and normoglycemic mice, results obtained at lower FT-IR frequencies were consistent with data reported in the literature. For example, the shoulder at 1707 cm^−1^, which is especially evident in the DNA spectral profile of the hyperglycemic mice, may be due to a guanine band shifted from 1717 cm^−1^ [21], [22]. The absorptivity at this spectral region may also indicates that the DNA samples analyzed here were not denatured [13], [20], [25], [26].

**Figure 6:**
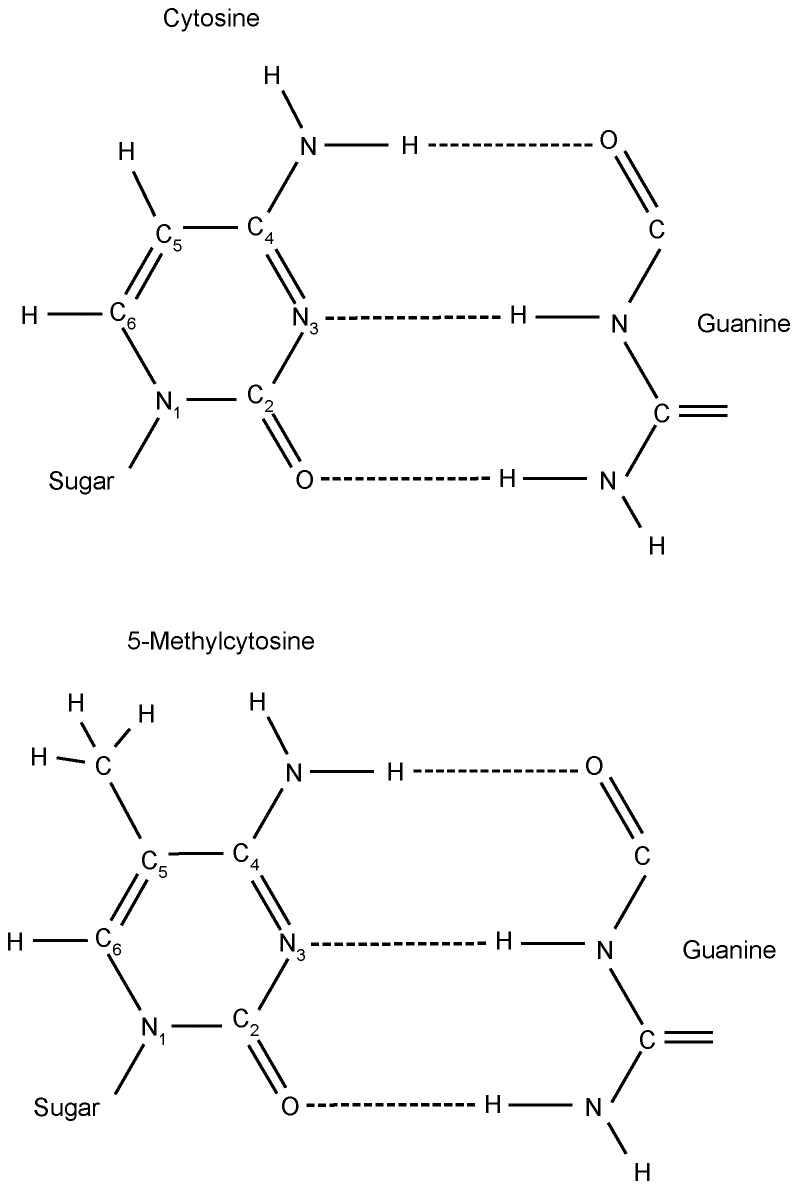
Molecular structure of cytosine (A) and 5-methylcytosine (B).

The peak at ∼1661 cm^−1^ in the normoglycemic mouse DNA shifted to 1650 cm^−1^ in the second-derivative spectra for the hyperglycemic NOD mouse DNA has been attributed to thymine [20]–[22] as well as an adenine peak [20], [22], also observed in an unpublished study by one of us (BCV). A peak in this spectral region has been observed in FT-IR spectral profiles from AT-biased DNA samples studied using an ARO [16]. In the NOD mouse liver cell DNA spectral profile, this peak may be due to pericentromeric heterochromatin satellite DNA, which contains AT-rich sequences [27]. On the other hand, according to Banyay and Gräslund [10], the upshift of cytosine ν C_2_ = O (Fig. 6) from 1652 to 1656 cm^−1^ may be “assigned to vibrational effects due to the presence of a 5-methyl group in cytosine”. The downshift detected herein from ∼1661 to 1650 cm^−1^ with decreasing DNA methylation in the hyperglycemic NOD mice may be supported by this proposition [10].

The band peak at 1493 cm^−1^, which is a more evident DNA peak in the hyperglycemic mice and is presumably assigned to cytosine only [16], may have resulted from an increase in unmethylated cytosine molecules for this DNA [7].

According to Taillandier and coworkers [*apud* 20], the PO_2_^−^ antisymmetric stretch frequency (1225 cm^−1^) is more sensitive to the DNA molecule geometry than the symmetric stretch (1088 cm^−1^). In the present investigation, a frequency change that affected the PO_2_^−^ vibrations from DNA with lower cytosine methylation involved symmetric rather than antisymmetric stretching. The DNA from hyperglycemic and normoglycemic mouse livers were obtained using the same protocol and examined under the same ambient relative humidity. Thus, if conformational changes were introduced in the DNA molecule, they were due to methylation status changes that result from the different mouse glycemic conditions [7]. According to Franklin and Gosling's report [28], at the relative humidity in which the DNA samples were examined (<75%), the DNA would acquire an A-conformation. However, at least in crystal structures, reports describe a transition between B-DNA and A-DNA that progresses through 13 conformational steps [29], [30] and demonstrated that methylated DNA sequences form standard A-DNA only if allowed to crystallize for a long period of time (2–3 months) [31]. DNA structures containing 5-methylcytosine would present local perturbations and even acquire a conformation with eccentric double helix characteristics that is neither B-DNA nor A-DNA (E-DNA); B-DNA transition to A-DNA would go through this intermediate DNA conformation [30], [31]. One study reported that a DNA molecule with both B-DNA and A-DNA was induced by cytosine methylation [29].

The absorptivity in the spectral region lower than ∼959 cm^−1^, which was more prominent for DNA from hyperglycemic mouse liver cell nuclei, is most likely associated with DNA O-P-O bending [32], indicating a less rigid DNA conformation with decreasing DNA methylation. Cytosine methylation has been reported to restrict DNA backbone bending and flexibility [33]. Interestingly, in hepatocyte nuclei from hyperglycemic NOD mice, chromatin becomes more unraveled compared with the normoglycemic controls [8].

The 900–790 cm^−1^ spectral region is also sensitive to the sugar conformation [10]. The peak at 839 cm^−1^ in the normoglycemic mouse DNA that apparently shifted to 836 cm^−1^ in the hyperglycemic mouse DNA may be related to changes in the sugar ring vibrations induced by lower cytosine methylation [10] and higher glycemic conditions.

In conclusion, changes in the NOD mouse liver cell cytosine methylation levels under hyperglycemic conditions, which alter DNA structures and chemical environment, were reflected in the corresponding FT-IR DNA signatures. The changes that affect cytosine methylation levels certainly interfere with DNA-protein interactions and, consequently, gene expression [10], [34], [35], which is expected for NOD hyperglycemic mouse liver cells [6].

## Materials and Methods

### Animals

Female NOD/Unib adult mice were obtained from the Multidisciplinary Center of Biological Investigation at the University of Campinas. The animals were reared under standard controlled conditions, fed extruded chow (Nuvital, Colombo, Brazil) and received water *ad libitum*. Their glycemia levels were measured through blood samples obtained by caudal puncture and subjected to weekly analyses by an automatic Accu-Check Active Performa glucose meter (Roche Diagnostic do Brasil, Jaguaré, Brazil) up to 24 h before they were killed. Glycemia levels within the range 90–100 mg/dL (5.00–5.55 mmol/L) were considered normal; glycemia levels >500 mg/dL (27.5 mmol/L) indicate severe hyperglycemia. The normoglycemic mice used as controls were matched by age to the hyperglycemic animals. The protocol involving animal care and use as well as chromatin analysis was performed in compliance with the Brazilian College of Animal Experimentation and approved (protocol no. 1608-1) by the Committee for Ethics in Animal Use of the University of Campinas (Brazil).

### Sample Preparation

**Figure 7:**
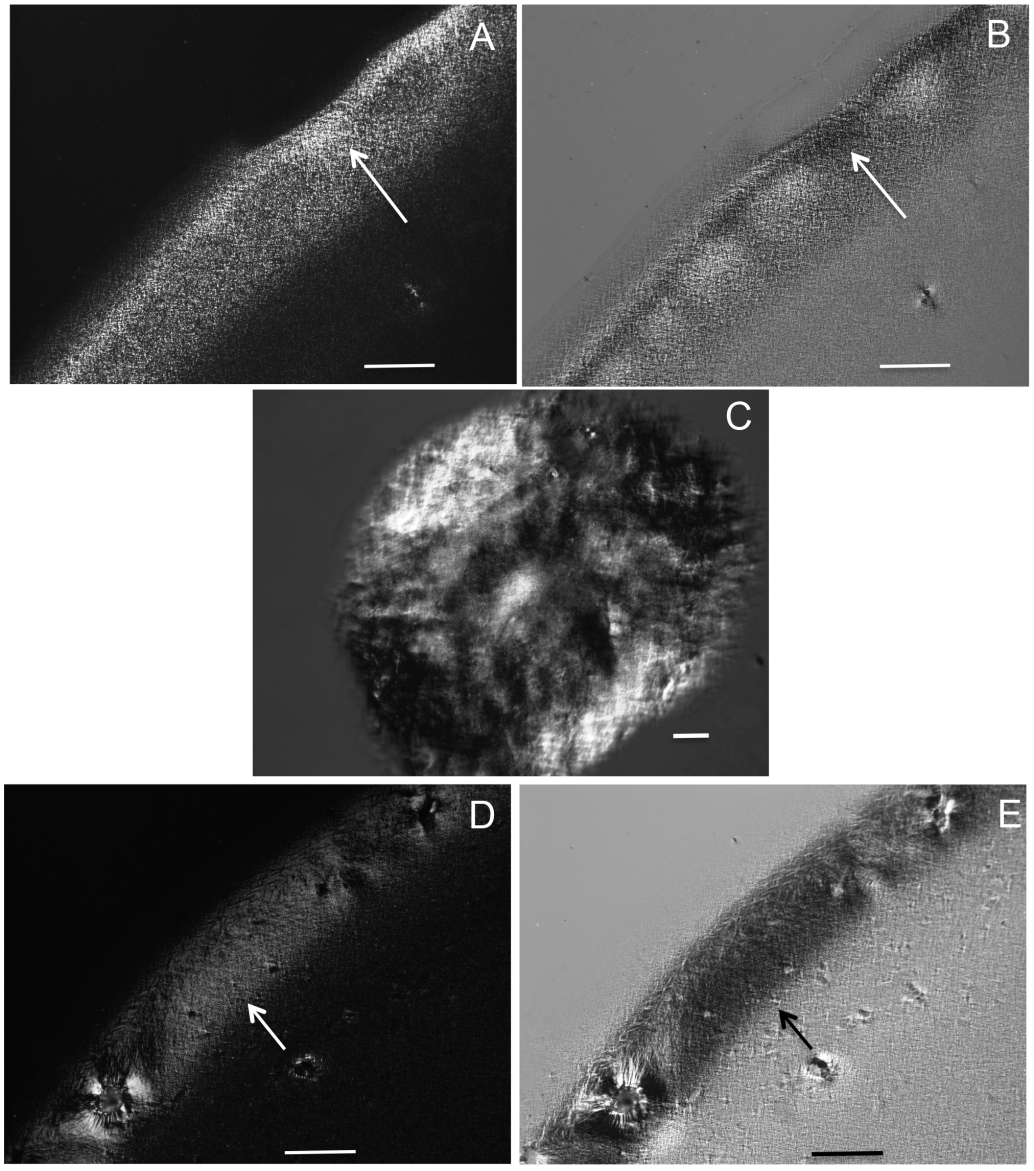
Optical anisotropy aspects of the DNA samples extracted for FT-IR analysis. Birefringence images are shown for the liver DNA from normoglycemic (A,B) and hyperglycemic (C-E) mice. Birefringence brilliance in the outer region of DNA drops dried on slides in A and D was compensated in B and E, respectively (arrows). The bars equal 100 µm.

A modified phenol-chloroform method [24] was used for DNA extraction from the isolated liver nuclei. Briefly, for each group, materials from three animals were assembled into one sample. Approximately 400 mg of DNA quantified by UV spectrophotometry were extracted twice with phenol-chloroform-isoamyl alcohol (25∶24∶1) and centrifuged at 14,000 rpm for 5 min at 4°C. The supernatant was then extracted once with chloroform-isoamyl alcohol (24∶1) and precipitated with 4 M sodium acetate at pH 5.0 in 2 volumes of absolute ethanol at −20°C overnight. Next, the samples were centrifuged at 14,000 rpm for 20 min at 4°C and washed in 70% ethanol. The 260/280 nm absorbance ratio (∼1.8) was obtained for the samples using the Thermo Scientific NanoDrop 2000 spectrophotometer (Wilmington, DW, USA) and indicates pure DNA according to the manufacturer. The extracted DNA was resuspended in a 0.9% NaCl solution and stored at −20°C until use. Next, it was washed in 80% ethanol to remove NaCl crystals, air dried and spread on gold-covered glass slides for FT-IR analyses. The phenol-chloroform protocol used does not affect DNA methylation patterns [36]. DNA samples that were prepared using the same protocol as for FT-IR but were spread on glass slides were examined for optical anisotropy in an Olympus BX51 polarization microscope (Tokyo, Japan). Birefringence with a negative sign demonstrated that the extracted DNA was in a helical double-stranded conformation (Fig.7). The ambient relative humidity at which the samples were examined for FT-IR and optical anisotropy was less than 75%.

### Equipment/Software

The DNA FT-IR spectral profiles were obtained using an Illuminat IR II™ microspectrometer (Smiths Detection, Danbury, USA) equipped with a liquid nitrogen- cooled mercury-cadmium-telluride (MCT) detector; an Olympus microscope; and Grams/AI 8.0 spectroscopy software (Thermo Electron Co., Waltham, USA). The performance validation of the equipment used a low signal-to-noise ratio (7929:1) [37]. The area for measurements was 50 µm per side; the sample and background absorbances were measured using 64 scans for each individual profile. Because an all-reflecting objective (ARO) has been especially recommended for FT-IR analyses of DNA vibrational properties in the 3600–3000 cm^−1^ spectral region [16], this type of microscope objective (16× magnification) was used here.

### Procedures

The spectral absorption signatures were obtained at wavenumbers ranging from 4000 cm^−1^ to 700 cm^−1^ with the spectral resolution of 4 cm^−1^. Ten spectral profiles were obtained for each sample. Baseline correction (using a four-point method and positioning the first point at ∼3700 cm^−1^) and normalization to the highest peak were performed for each spectral profile; an average profile was then calculated for each sample. For the peak fitting procedure using a Gaussian function a low sensitivity level was applied to the 2987–2849 cm^−1^ spectral region using the same software. In accordance with the software instructions, the area for the selected bands was calculated using the trapezoidal rule of integration and the center of mass was defined as the X coordinate of the point where the peak areas are equal on either side. To confirm the frequency positions of the peaks and differences in peak intensities at specific spectral windows, Savitzky-Golay's second-derivative spectra were also obtained [19], [38].
